# A psychophysical performance-based approach to the quality assessment of image processing algorithms

**DOI:** 10.1371/journal.pone.0267056

**Published:** 2022-05-05

**Authors:** Daniel H. Baker, Robert J. Summers, Alex S. Baldwin, Tim S. Meese

**Affiliations:** 1 Department of Psychology and York Biomedical Research Institute, University of York, York, United Kingdom; 2 College of Health & Life Sciences, Aston University, Birmingham, United Kingdom; 3 Department of Ophthalmology & Visual Sciences, McGill Vision Research, McGill University, Montreal, Quebec, Canada; Justus Liebig Universitat Giessen, GERMANY

## Abstract

Image processing algorithms are used to improve digital image representations in either their appearance or storage efficiency. The merit of these algorithms depends, in part, on visual perception by human observers. However, in practice, most are assessed numerically, and the perceptual metrics that do exist are criterion sensitive with several shortcomings. Here we propose an objective performance-based perceptual measure of image quality and demonstrate this by comparing the efficacy of a denoising algorithm for a variety of filters. For baseline, we measured detection thresholds for a white noise signal added to one of a pair of natural images in a two-alternative forced-choice (2AFC) paradigm where each image was selected randomly from a set of *n* = 308 on each trial. In a series of experimental conditions, the stimulus image pairs were passed through various configurations of a denoising algorithm. The differences in noise detection thresholds with and without denoising are objective perceptual measures of the ability of the algorithm to render noise invisible. This was a factor of two (6dB) in our experiment and consistent across a range of filter bandwidths and types. We also found that thresholds in all conditions converged on a common value of PSNR, offering support for this metric. We discuss how the 2AFC approach might be used for other algorithms including compression, deblurring and edge-detection. Finally, we provide a derivation for our Cartesian-separable log-Gabor filters, with polar parameters. For the biological vision community this has some advantages over the more typical (i) polar-separable variety and (ii) Cartesian-separable variety with Cartesian parameters.

## 1. Introduction

Quality is difficult to assess. Suppose you are handed a picture (a photograph, a print, or an electronic image) and asked for your opinion about image quality. What do you judge? You know the task is subjective, and you might have opinions about various aspects of the image: saturation, contrast, lightness, shadows, blur, definition, image noise and other artefacts. But which are more important, how are you going to quantify these properties, how are you going to combine them, and who is to say that your opinion is the one that counts?

These issues are well known in the image processing community, and several approaches have been adopted, including automated numerical procedures [e.g. 1], some of which are based on the properties of human vision [e.g. 2,3] (see [4] for a review). However, the general view is that the “gold standard” is a human observer [5] which means that human judgements are required. This is sometimes done using a rating scale. There are several variants including moving a slider [6,7] and making numerical comparisons of a processed image against a standard one [7], but in many cases the reader is simply invited to judge an unsupported claim by the author that the subjective quality of one particular method is the best [e.g. 8,9]. Even when results from human ratings are provided, the subjective nature of the approach means the criterion sensitive problems outlined above remain.

The problem we tackle here is how to derive an objective and quantifiable human quality measure of image-processing algorithms. Taken literally of course, this cannot be done. Image quality is inherently subjective—if it was quantifiable, it would be called *image quantity*. Nonetheless, this has not prevented previous attempts to map subjective experience onto numbers as described above. However, our approach is different. Instead of using criterion-sensitive judgements as a proxy for quality, we use perceptual performance. Our logic is that if an observer cannot discriminate one image from another, the images cannot differ in perceptual quality. The signal level at which a change in image quality can be detected is sometimes called the just noticeable difference (JND) and is not without precedent in the image processing literature [10,11]. In Section 1.2 we develop this idea for a denoising algorithm by way of illustrating the approach. In Section 4.4 of the Discussion, we consider possible developments for testing other types of image-processing algorithms.

### 1.1 Denoising

**Fig 1:**
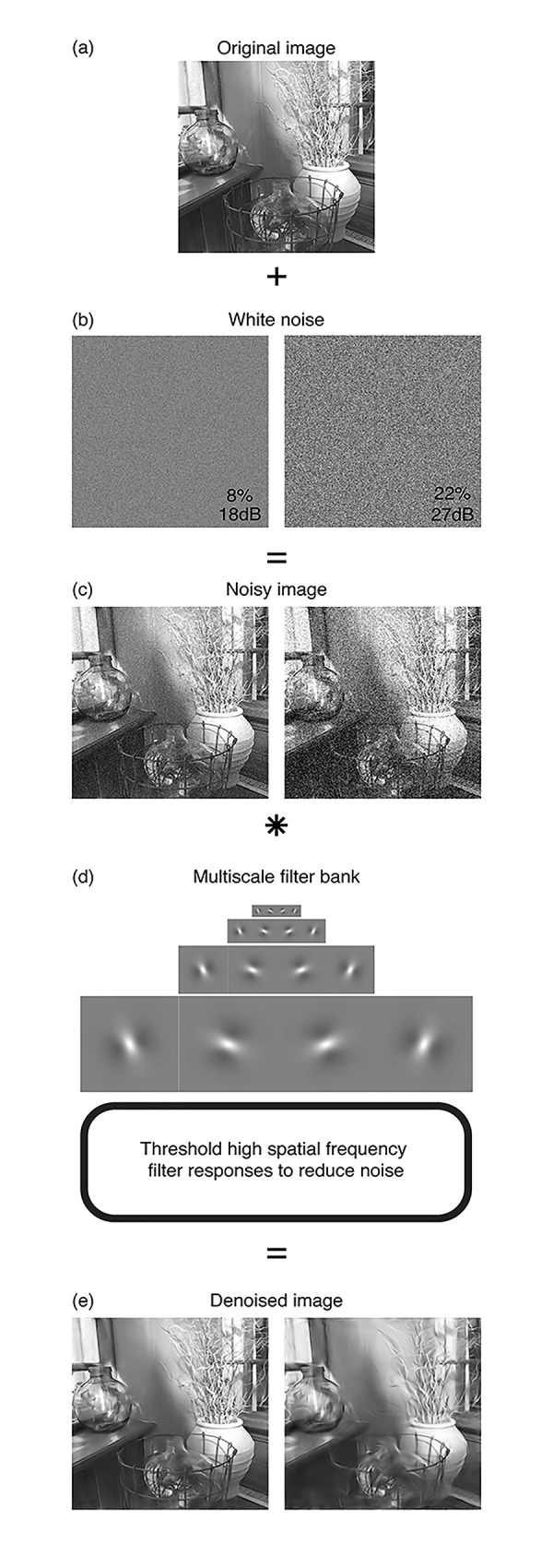
Illustration of an image denoising technique. White noise (b) is added to an image (a) to produce a noisy image (c). The image is then decomposed by convolution with a bank of filters (d) at different orientations and spatial scales (only a subset of filters is shown here). High frequency responses are then thresholded to reduce the amplitude of the noise, producing a final denoised image when the representation is transformed back to the spatial domain (e). The process is shown for two levels of noise: Pixel standard deviations of 8% (left columns) and 22% (right columns). For the more severe noise, there are visible ringing artefacts from the filtering in the denoised image (right image in part e). The example image used here was not part of the image set used in the experiments. Larger examples are provided in S1 File.

The removal of unwanted noise from digital images is a task for which numerous algorithms have been proposed (e.g., [12–14], see [15] for a recent review). Many denoising schemes [12,13] take a multi-scale filtering approach [see 16], inspired by the architecture of the human visual system [17]. These techniques work by decomposing the image into discrete bandpass signals by filtering in the Fourier domain (or, equivalently, by convolution). Filters containing large amounts of noise (typically those at the smallest spatial scales) are thresholded. For example, filter responses with small amplitudes are likely to be noise and based on a threshold amplitude, these are either reset to zero (hard thresholding) or attenuated (soft thresholding). The image is then reconstructed from the remaining components, resulting in improved quality (see Fig 1).

**Fig 2:**
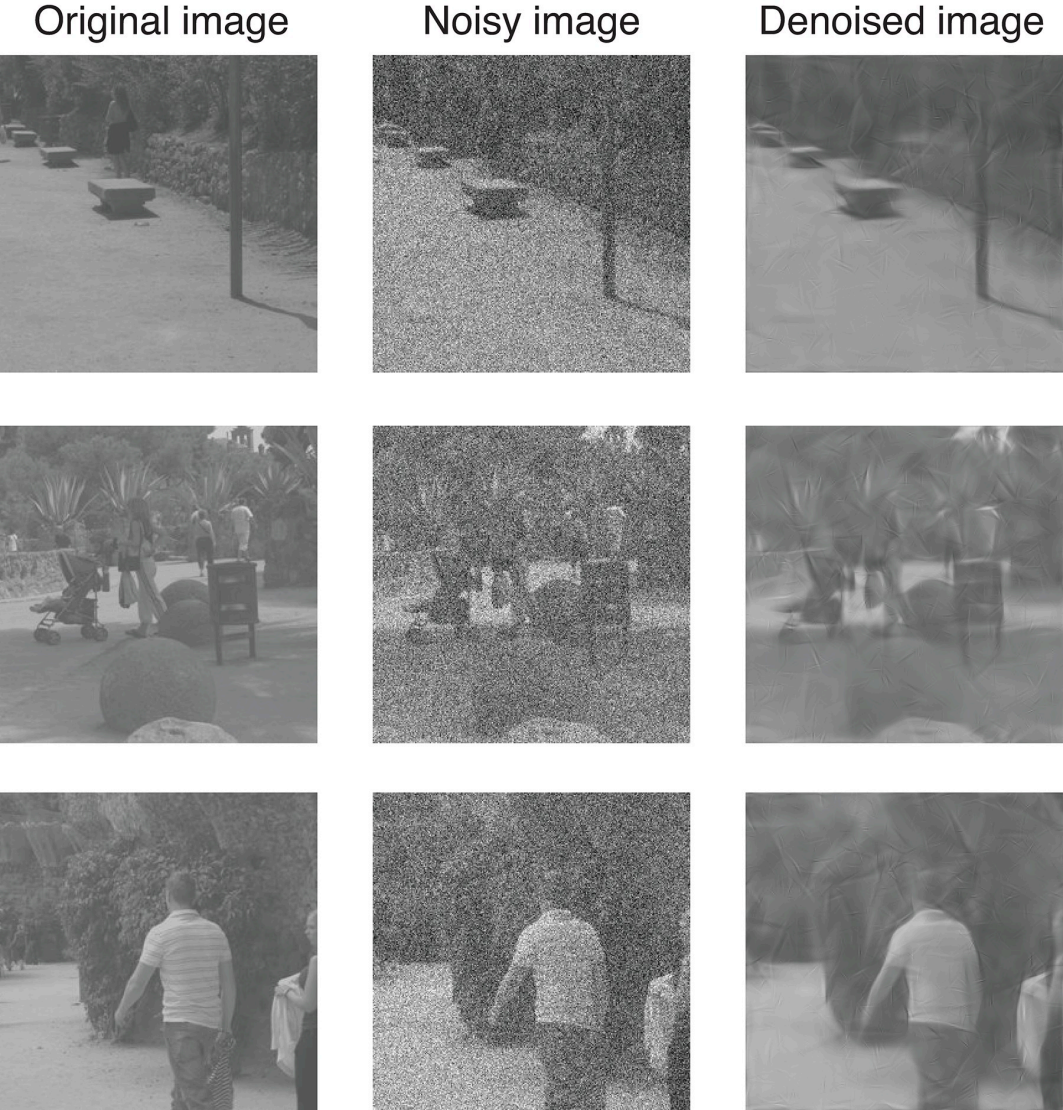
Example images to illustrate denoising. The left column shows a set of images used in the experiments with no noise added. The middle column shows the same images with added white noise, and the final column shows the images output by a denoising algorithm. Note that for the purposes of illustration, the noise level shown is substantially above the detection thresholds measured in the experiments.

Such image denoising techniques are not perfect. For large amounts of noise, the denoised image will contain noticeable ringing artefacts from the filtering (see Fig 2). Essentially, these are copies of the filter kernels that were driven above the algorithm’s cutoff threshold by the noise. Furthermore, because of the thresholding at high spatial frequencies, legitimate image information at fine spatial scales will be lost, so the denoised image might appear blurry (i.e., low pass filtered). This means there will be a limited range of noise levels over which denoising produces acceptable images without noticeable distortions.

The success of a given algorithm and/or filter type is usually assessed numerically, by calculating the mean difference between pixels in the original and denoised images, often the peak signal-to-noise ratio (PSNR). Although error statistics of this type permit direct comparisons between algorithms, they have several drawbacks (as discussed more generally by [18]). First, the original image is necessary for the calculation, limiting the usefulness of error statistics to cases where noise is added artificially. In many applications, such as removing sensor noise from a digital photograph, there is no noise-free original image with which to perform the calculation. Second, this method does not measure human perceptions of images (e.g., the salience of blurring or filter artefacts) and rankings of simple error statistics and human perception can be inconsistent [2]. Third, because different algorithms and filters produce different artefacts, it is possible for two denoised images with the same numerical quality score to differ in their perceived quality. Fourth, we are unaware of any previous work to assess the human perceptual quality of denoising algorithms. All this points to a need for a reliable method of assessment to be developed.

### 1.2. Subjective perceptual measures and objective measures of performance

Wherever possible, experiments on visual perception avoid the vagaries of rating scales for the reasons of criterion-sensitivity outlined at the beginning of this section. In fact, many experiments in visual psychophysics fall into one of just two broad categories. 1. Measures of a point of subjective equality. 2. Measures of performance. The first is subjective, by definition, and involves making a comparison between two images where a property of one is adjusted until a perceptual match is achieved along the dimension of interest. Could such an approach be used to assess image quality? One problem here is in knowing what to ask observers to judge. Being asked to compare ‘quality’ is fraught with problems as outlined already though it has been tried, seemingly with some success [19]. Alternatively, one might pick a more concrete dimension—perceived global contrast for example [e.g. 20] but there are two obvious problems. First, this misses every other image property that might be involved in the perception of quality. Second, in any case, it is not clear how finding the point where one image looks, in some sense, like another along a dimension of interest, can tell the investigator anything valuable about the image-processing algorithm under scrutiny unless one of the image sets is already quantified for human perception, but this is precisely the problem being addressed.

Fortunately, the second approach above offers much more promise. A typical experiment uses two-alternative, forced-choice, where observers are required to detect a target signal present in one image but not in another. The experimenter then plots percent correct (for there is ground truth) as a function of signal strength to derive the observer’s sensitivity to the signal (the reciprocal of the signal strength corresponding with some criterion level of performance, such as 75% correct). This approach provides a criterion-free measure of signal sensitivity. Or put another way, it provides an objective tap on human perception, but avoids the subjectivity inherent in the first approach (though see Section 4.2) and with the inherent vagaries of rating scales.

Here, we develop and test a simple psychophysical forced-choice technique designed to achieve the requirements above. It is inspired by masking experiments [e.g. 21] where an observer’s ability to detect a target (here the added image noise) is impaired by some treatment (typically a masking stimulus, but here, the application of a denoising algorithm). By measuring detection thresholds for (i) noise added to the original image, and (ii) noise added to an image that subsequently passes through a denoising algorithm, it is possible to determine the points at which (i) added noise and (ii) filter artefacts become visually salient. (i.e. the point at which degradation of image quality becomes detectable). Comparing these two thresholds (expressed in common units of the added noise) indicates the amount of added noise an algorithm conceals from the human visual system. To illustrate our method, we used a single denoising algorithm [12] and compared two filter types that are relevant to the vision community and described in the following section.

### 1.3. Log-Gabor filter design

Log-Gabor filters are widely used in image processing applications [e.g. 22] including image denoising [12,13]. First proposed by Field [23], they are defined in the Fourier domain, and have a frequency response that is Gaussian on a log-spatial frequency axis. This is combined with an orientation component typically defined in polar coordinates (e.g., the filter energy falls off as a function of polar angle). In many applications, log-Gabor filters are preferred over more traditional (linear) Gabor designs, because they have the property of being D.C.-balanced for any phase of filter. This derives from the log spatial frequency tuning which prevents the filter from including energy at zero frequency.

**Fig 3:**
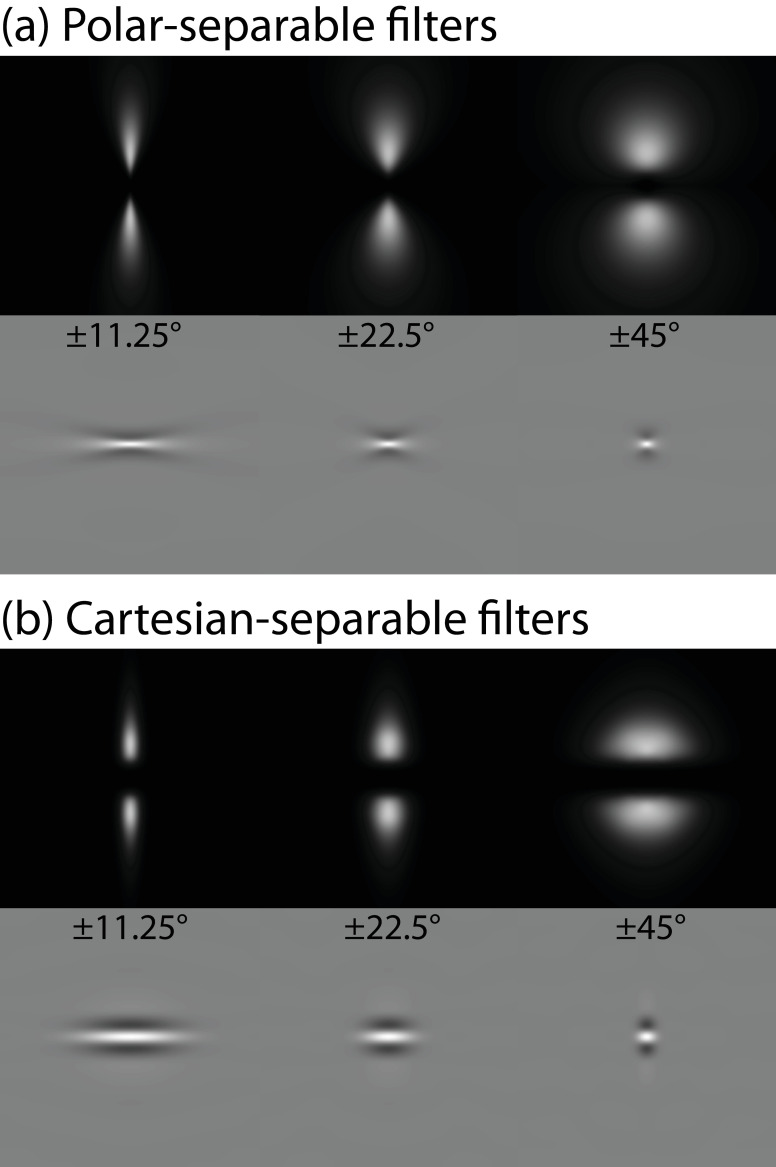
Example log-Gabor filters of different orientation bandwidths for (a) polar-separable and (b) Cartesian-separable filters (see text for details). In each panel, the upper row shows the Fourier amplitude spectra, and the lower row the cosine-phase spatial transform (i.e. the filter kernel). All filters have a spatial frequency bandwidth of 1.43 octaves, and orientation bandwidths (*h* in Eq 4) of (from left to right) ±11.25°, ±22.5° and ±45°. Note how the filter kernels of these narrowband polar filters (lower row, a) splay outwards, whereas the equivalent Cartesian filter kernels do not (lower row, b).

Example Fourier spectra for three polar-separable log-Gabor filters with different orientation bandwidths are shown in the upper row of Fig 3A, with their filter kernels shown by the cosine-phase spatial transforms in the lower row. The filter kernels have a superficial similarity to linear Gabor patches (the product of a sinusoidal carrier and a Gaussian envelope). Polar-separable log-Gabor filters like these are used widely [e.g. 12,13], but are inconsistent with the filter properties of human spatial vision [24,25]. Furthermore, a property of polar-separability is that for narrow orientation bandwidths the spatial profile of the filter kernel splays outwards (see Fig 3A, lower left). We have never seen any evidence for this in the biological vision literature and wondered whether the curious shapes of these filter kernels might produce particularly salient artefacts when used with a denoising algorithm.

An alternative to polar-separable filters is to define the orientation component in Cartesian coordinates instead [26]. Previous studies [e.g. 22] have expressed the bandwidths of such filters in terms of the orthogonal (*u*, *v*) axes of the Fourier plane. However, in the biological vision literature at least, orientation and spatial frequency (polar) terms are usually preferred, even if these are not the separable dimensions.

We therefore define Cartesian-separable filters by the equation:

logGab2D(f,θ)=logGab1D(f,θ)×orthFunc(f,θ), (1)

where (*f*,*θ*) are polar coordinates in the Fourier plane (spatial frequency (in cycles per image or cycles per degree) and orientation (in degrees)). The first function is defined as:

$$logGab1D(f,\;\theta )=exp\left[\frac{-{\left\{{log}_{2}(\frac{f|cos(\theta -\theta_{0})|}{f_{0}})\right\}}^{2}}{2(0.424{\omega )}^{2}}\right]$$ (2)

where *f*_0_ and *θ*_*0*_ are the centre (peak) spatial frequency and orientation of the filter, and *ω* is the spatial frequency bandwidth (full-width at half-height) in octaves.

The second function is defined as:

$$orthFunc(f,\;\theta )=exp\left(\frac{-{\left\{fsin(\theta -\theta_{0})\right\}}^{2}}{2\eta^{2}}\right),$$ (3)

where

$$\eta =f_{0}sin(h)\sqrt{\frac{1}{ln4-{\left(\frac{{log}_{2}|cos(h)|}{0.424\omega }\right)}^{2}}},$$ (4)

and where *h* is the orientation bandwidth in degrees. The derivation of these equations is presented in Appendix A, with example Matlab code in Appendix C. For comparison, we also used standard polar-separable filters. These are defined in a similar manner, with the two terms in Eq 1 given by:

$$logGab1D(f,\;\theta )=exp\left(\frac{-{\left\{{log}_{2}(\frac{f}{f_{0}})\right\}}^{2}}{2{log}_{2}({\omega )}^{2}}\right),$$ (5)

where

$$orthFunc(\theta )=exp\left(\frac{-{\left|atan2\{sindiff,\;cosdiff\}\right|}^{2}}{2h^{2}}\right),$$ (6)

where sindiff = sin(*θ*)cos(*θ*_*0*_)–cos(*θ*)sin(*θ*_*0*_), cosdiff = cos(*θ*)cos(*θ*_*0*_) + sin(*θ*)sin(*θ*_*0*_) and all terms retain their previous meanings. The term atan2 is a two-argument arctangent function, which returns the angle between the origin and the x-axis.

Cartesian-separable filter kernels do not splay outwards at narrow orientation bandwidths, giving them a more streamlined spatial profile (Fig 3B), but retaining the D.C.-balance of their polar-separable cousins. We have used them successfully in several image processing models [e.g. 26–29] and as a psychophysical stimulus [30]. They are also consistent with psychophysical masking studies [24,25] and single-cell physiology of the primary visual cortex [31].

However, there is one limitation in this filter design. As orientation bandwidth increases, the ‘orthogonal’ Fourier profile becomes more elongated. This means

that unlike the polar filters (for which the spectrum eventually completes as a ring for a fully isotropic filter), Cartesian-separable filters have a maximum possible orientation bandwidth. Through numerical analysis (Appendix B) we determined that this upper bound depends solely on the spatial frequency bandwidth, such that, to a close approximation, *h*_*MAX*_ = 15 log_2_(*ω*)+45 deg, for bandwidths of 0.7 < *ω* < 5 octaves. Fortunately, it is unusual to require orientation bandwidths outside this range in practice.

## 2. Materials and methods

### 2.1 Apparatus and stimuli

**Fig 4:**
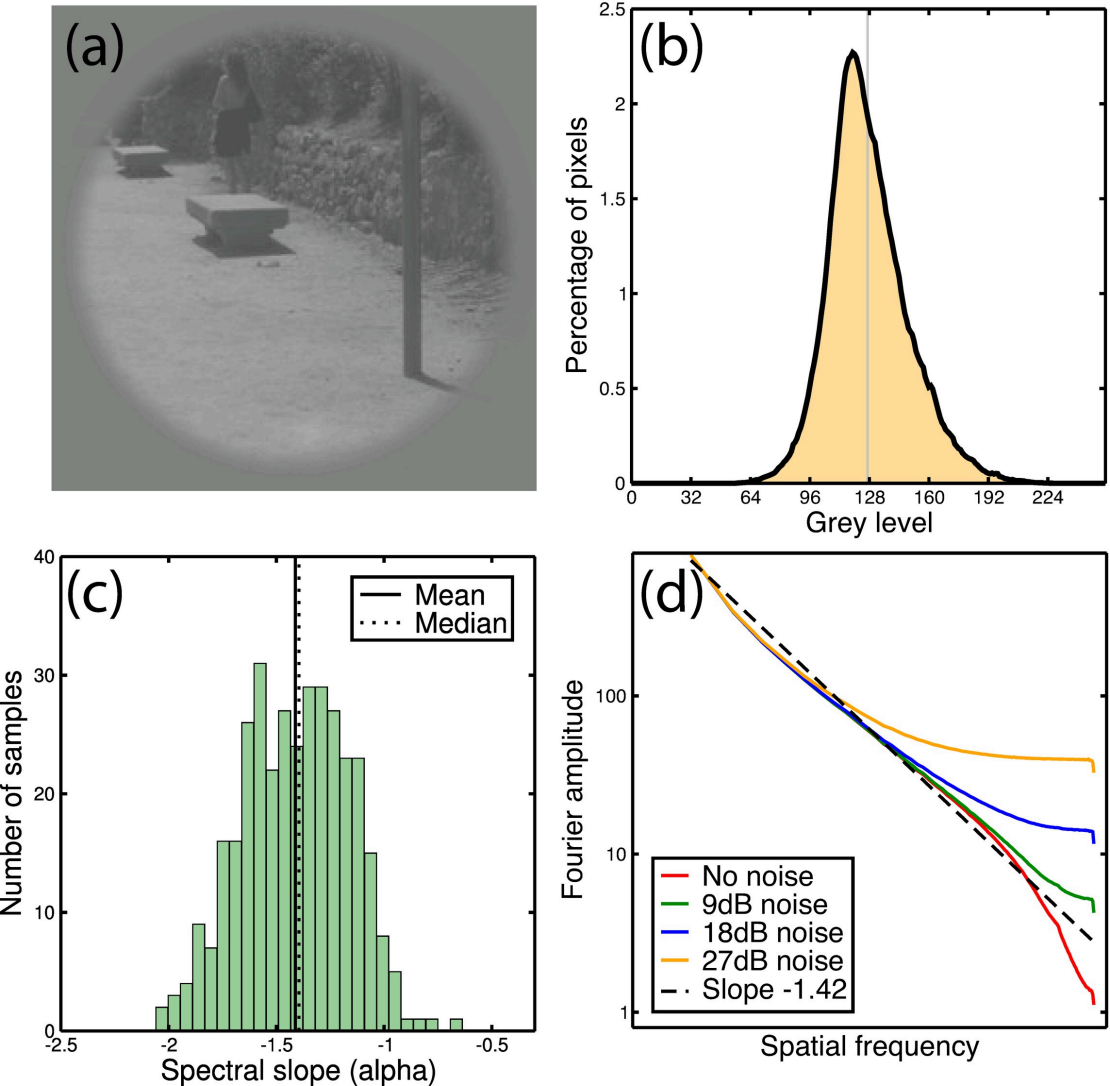
Example image, and statistics of image set. Panel (a) is an example windowed image used in the experiment, shown here with no added noise. Panel (b) shows the grey level histogram for the DC-balanced image set (308 images, each 256x256 pixels). The distribution shows slight positive skew, so the modal grey level (119) is below the mean (128). Panel (c) is a histogram showing the frequency of different spectral slopes in the image set. The mean and median alpha values are around -1.45. In panel (d), the effect on the spectral slope of adding white noise is shown. Values are averaged across all 308 images in the data set. Increasing amounts of white noise distort the approximately linear (on log-log axes) slope obtained with no added noise (red curve). With large amounts of white noise, the spectrum is flat (a slope of 0) over a substantial portion of the frequency range (e.g., for noise of 27dB, orange curve).

We used images (e.g., Figs 2 and 4A) from the Barcelona Calibrated Image Database (described in [32], and downloaded from http://www.cvc.uab.es/color_calibration/). The database consists of 350 images of natural and man-made environments around Barcelona, taken with a single camera. Crops of 256x256 pixels were extracted from the lower right corner of each image and converted to greyscale (our choice of crop guaranteed that a grey sphere intended to aid colour calibration was excluded from each image). We D.C.-balanced each image and reduced its contrast by a factor of two to permit noise to be added with no clipping (Fig 4B). We assessed the spectral slope of each image by fitting a linear function to the binned amplitude spectrum across 100 equally spaced frequency bins, pooling over orientation. We rejected 42 images with exceedingly steep or shallow slopes (often these were out of focus images). This left 308 images, with a mean RMS contrast of 21dB and a mean spectral slope (see Fig 4C and 4D) of α = -1.45, within the range reported previously for ensembles of natural images [23,33–36]. The grey level histogram showed slight positive skew, as is typical of natural images [37].

We added various amounts of 2D white pixel noise to the image stimuli, using a unique noise sample for each image and noise level. The noise level was defined as the RMS contrast (e.g., the standard deviation of pixel values) expressed in decibels (dB), such that *C*_*dB*_ = 20 log_10_(*C*_*RMS*_), where *C*_*RMS*_ is the RMS contrast expressed as a percentage. We then used a denoising algorithm to remove the noise. The algorithm is described in detail by Fischer et al. [12], and a Matlab implementation is available online (http://www.iv.optica.csic.es/resources/Software-deployment/Denoising-Log-Gabor/log_gabors_deno.zip). Note that the algorithm requires a threshold parameter to be set manually by the user. For a given filter bank, we determined optimal thresholds for each noise level based on fitting a polynomial function to the mean PSNR statistic across images as a function of threshold. These threshold values were then applied to all images with that noise level. This ensured that the algorithm performed as well as was reasonably possible for a given set of images. For baseline conditions (no added noise), we used the threshold consistent with an added noise contrast of 1% (0dB). This produced PSNR values far higher than the detection thresholds we subsequently measured, implying that any artefacts introduced by the denoising algorithm must be invisible for this condition (which we verified by visual inspection).

Both Cartesian-separable and polar-separable log-Gabor filters (see above) were used in different experimental conditions. All filters had a spatial frequency bandwidth of 1.43 octaves, and were generated at 6 spatial scales, in octave steps. We compared different orientation bandwidths for the filters, with full-width-at-half-heights ranging from 11.25° (16 filters per spatial scale) to 45° (4 filters per spatial scale). Finally, all stimuli were multiplied by a circular raised cosine envelope (e.g., a central plateau with a blurred edge) with a full-width-at-half-height of 240 pixels (see Fig 4A).

All stimuli were displayed on a Nokia MultiGraph 445x monitor which was Gamma corrected using standard techniques and had a mean luminance of 60*cd/m*^*2*^. The monitor was viewed from 60cm, producing a resolution at the eye of 24 pixels per degree of visual angle, such that the windowed stimuli had a full width at half height of 10 degrees. We used a ViSaGe framestore system (Cambridge Research Systems Ltd., Kent, UK) to store the stimuli and control presentation.

### 2.2 Procedure

Observers were seated in a darkened room, with their head in a chin rest at the appropriate viewing distance (60cm). A fixation dot was present throughout in the centre of the display. On each trial two stimuli were presented sequentially at fixation for 100ms each, with an interstimulus interval of 400ms. The stimuli were different images from the set, selected at random with the restriction that any given image would appear only once during a block of trials.

We used different images in the pair to encourage global perceptual judgements of the two images. This reflects our general view that image quality refers to the entire image and that this is what should be judged. In contrast, if we had used the same images within each pair, then observers might have locked onto local differences in the rendering of image features (for example the shirt in the lower row of Fig 2). While this is a legitimate psychophysical task, it is less clear (to us) that it pertains to the perception of image quality. However, such an approach might be of value if, for example, local image processing was the object of enquiry. In pilot work, we ran the experiment on one observer using this protocol. Thresholds were slightly lower (1.8dB on average) than for the main experiment, but results were otherwise unchanged.

For the baseline condition, one image had noise added (but did not pass through the denoising algorithm), and the observer’s task was to indicate which interval contained the noise [e.g. 38] using the buttons on a mouse. Auditory feedback indicated correctness of response.

In the denoising conditions, a pair of images (one with added noise, the other without) were passed through the denoising algorithm on each trial before being displayed. As before, the task was to detect the image that contained the noise. The denoising algorithm had no visible effect on the no-noise image, and it attenuated noise in the other image. Thus, practically speaking, the task in these conditions was to detect the distortions (filter artefacts and blurring) caused by the denoising algorithm on the image with added noise (see Fig 2).

There was one baseline condition and six (2 filter types × 3 orientation bandwidths) denoising conditions, each of which was repeated four times by each observer. The conditions were run in separate blocks, carried out in random order, and taking around 3 minutes each to complete. For each block, a pair of 3-down-1-up staircases [39] determined the level of noise added on each trial, spaced in steps of 3dB (factors of √2). We fitted cumulative log-Gaussian functions to the psychometric (% correct) data from each block to estimate thresholds in logarithmic (dB) units of added noise contrast. These thresholds were averaged across repetitions.

### 2.3 Observers

Three observers completed the experiment. DHB and RJS were both authors and therefore aware of the purpose of the experiments. Observer ASB was psychophysically experienced, but naïve regarding the experimental hypotheses at the time he participated, and subsequently became a co-author. Observers wore their normal optical correction if required and had no known visual abnormalities. Observer DHB was tested at some additional intermediate filter bandwidths not shown to the other observers.

### 2.4 Ethics statement

Procedures were approved by the ethics committee of the School of Life and Health Sciences at Aston University (approval number #856). Participants were the first three authors of the study, and provided written informed consent before participating.

### 2.5 Data availability statement

Raw psychophysical data from the experiment reported here are available at: http://doi.org/10.17605/OSF.IO/EDTU7

## 3. Results

**Fig 5:**
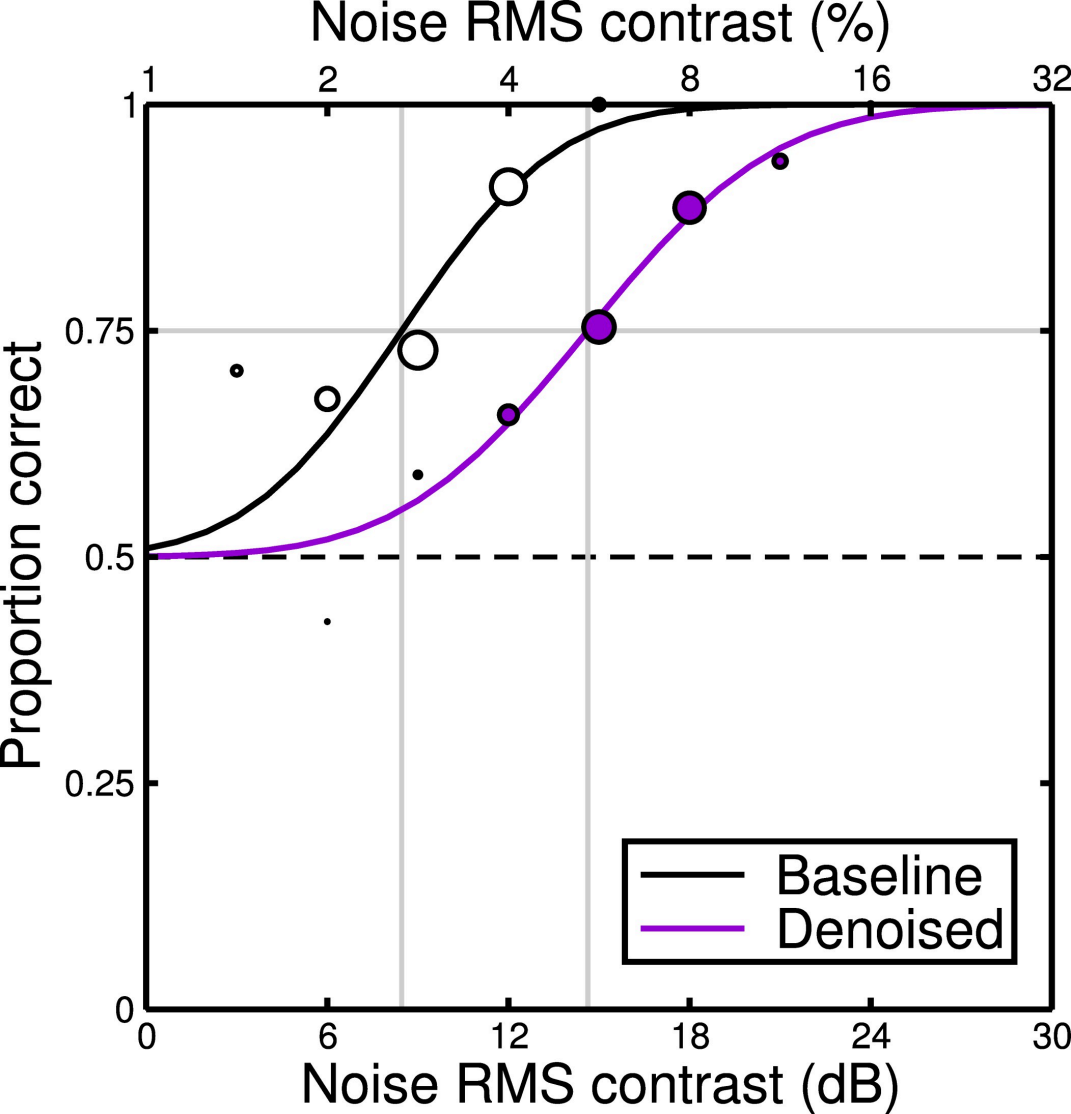
Example psychometric functions for observer DHB. Data points represent the proportion of trials on which the observer correctly identified the stimulus with added noise. The guess rate of 0.5 is given by the horizontal dashed line. Symbol size at each level is proportional to the number of trials. Curves are fits of the cumulative log-Gaussians, with vertical grey lines indicating the noise contrast at threshold performance (75% correct). In the Baseline condition (white symbols) the target was white pixel noise added to one of the images in the 2AFC pair. In the Denoised condition (purple symbols) the stimuli were the from the same set as at baseline, but after being passed through the denoising algorithm.

Example psychometric functions for two conditions and one observer are shown in Fig 5. They show the proportion of correct responses at each noise contrast level. The psychometric functions are monotonic and sigmoidal, typical for detection tasks. Their slopes are similar across the baseline and denoising conditions. Cumulative log-Gaussian functions provide a good fit to the results, and ‘threshold’ was taken to be the signal level corresponding with a performance level of 75% correct. Each threshold was estimated from four repetitions of a condition, which took around 3 minutes each.

**Fig 6:**
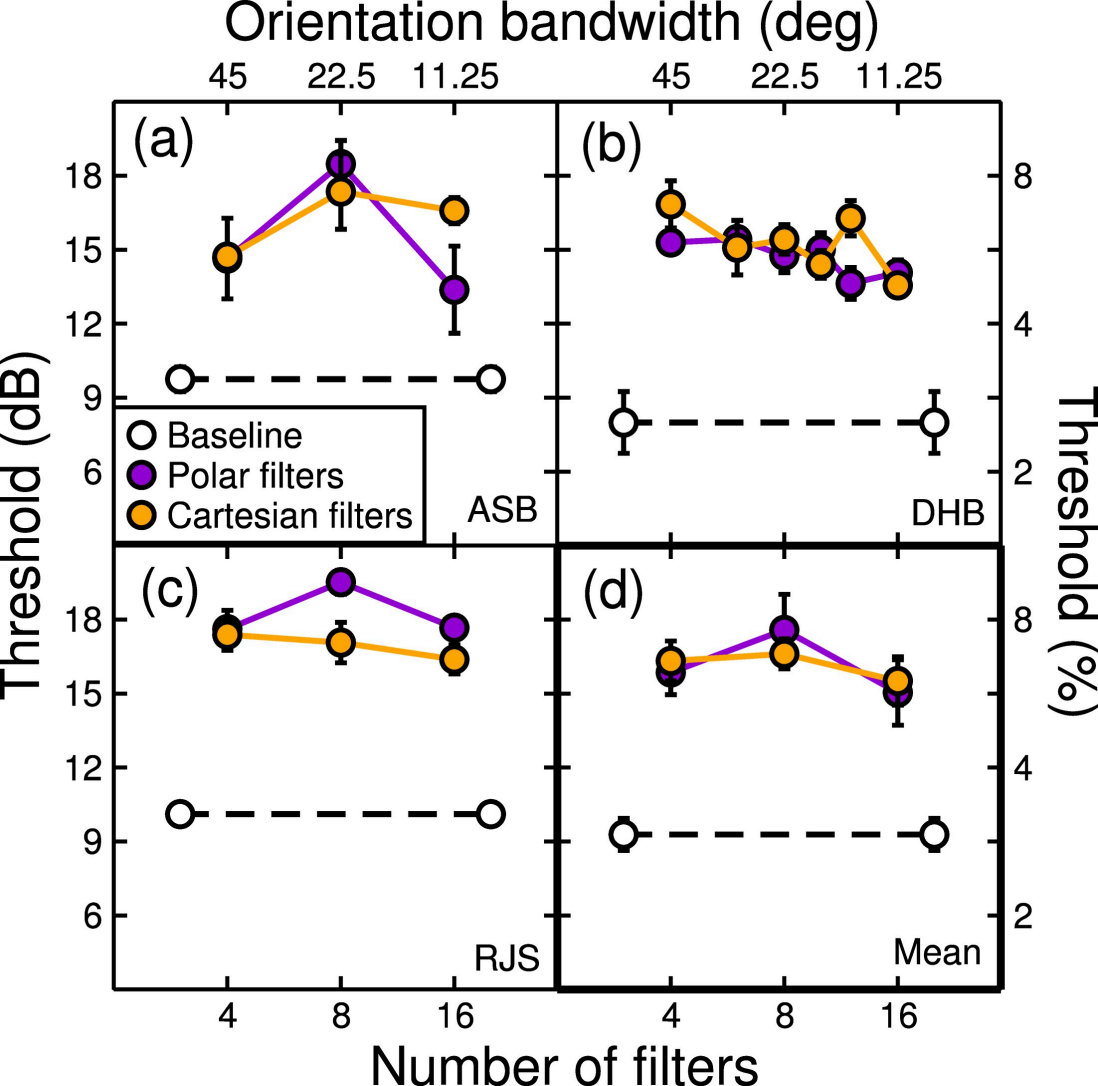
Thresholds for individual observers (a-c) and their average (d). Each point is the average of four repetitions (a total of around 420 trials) of the experiment (a-c) or three observers (d), with error bars giving ±1SE of the mean. White symbols are contrast thresholds for detecting which of a pair of images had white noise added to it. Coloured symbols are thresholds for the same task, but after passing all images through a denoising algorithm. Thus, the difference between white and coloured symbols indicates the amount of visible noise removed by the algorithm, which on average was around 6dB.

Thresholds for three observers and their average are shown in Fig 6. In all cases, the baseline thresholds (white circles) were substantially lower than the thresholds for denoised stimuli (orange and purple symbols). This threshold elevation by the denoising algorithm means it successfully ‘hid’ some of the added noise, rendering it undetectable. Averaging across different observers and filter types, the denoising algorithm increased threshold by 6dB. In other words, the algorithm concealed twice as much noise as could be detected by the human visual system prior to denoising. To our knowledge, this is the first time a performance-based measure of denoising has been reported.

Comparisons between filter orientation bandwidths, and between filter types do not reveal significant effects at the group level (two-way ANOVA on thresholds, all *p* > 0.05). There are some notable features within observers, such as greater threshold elevation for ASB at intermediate filter bandwidths (Fig 6A), and a slight downward trend to the functions for DHB (Fig 6B). However, these were not consistent across observers, and do not lead us to general conclusions. On average (Fig 6D), our novel psychophysical analysis shows that this particular algorithm is robust to changes in filter bandwidth, and that both polar- and Cartesian-separable filters are equally effective for image denoising.

### 3.1. Comparison with error statistics

A more traditional method for evaluating denoising algorithms is to calculate an error statistic, such as the mean squared error (MSE)

$$MSE=\sqrt{\frac{\sum ({image}_{0}-{image}_{N}{)}^{2}}{n}},$$ (7)

where image_O_ is the original image (scaled from 0–1), image_N_ is the image with noise added, and *n* is the number of pixels.

From this, one can then calculate the peak signal-to-noise ratio (PSNR; e.g., [18])

$${PSNR}_{dB}=10\times {log}_{10}\left(\frac{L^{2}}{MSE}\right),$$ (8)

where *L* is the dynamic range, with a value of 1 for our images (scaled 0–1). Notwithstanding the problems with this statistic, as outlined in the Introduction (Section 1), we wondered how well it would fare for the images used here and how it would compare with previous studies.

**Fig 7:**
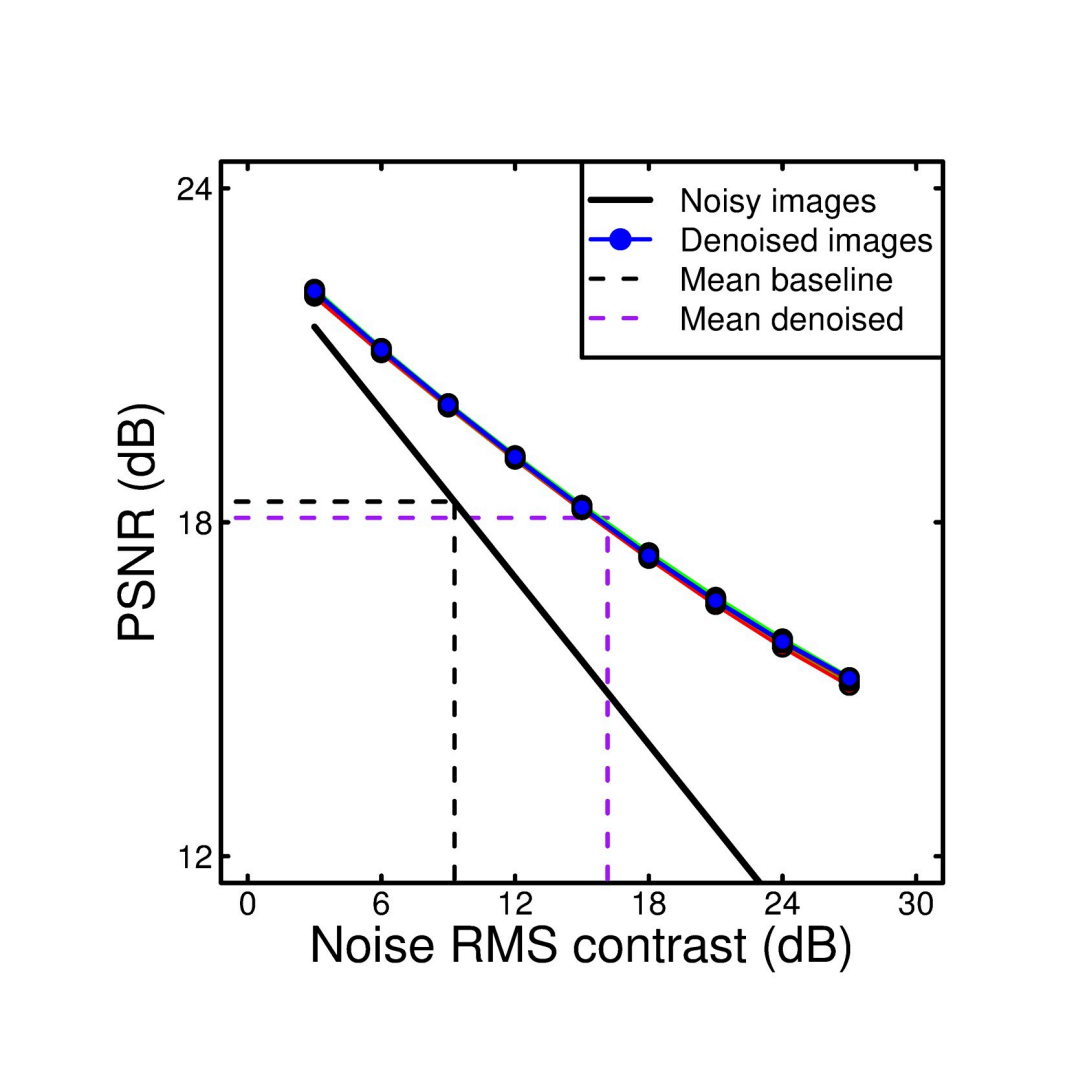
PSNR values for noisy (black line) and denoised (circles) images, calculated using Eq 8. Differences between filter types and bandwidths (different coloured circles) were negligible. The dashed lines project the mean baseline (black) and mean denoised (purple) thresholds from Fig 6D onto the PSNR scale. Observers reached threshold at very similar PSNR levels for the noisy and denoised images.

The PSNR can be calculated for images with added noise, and the same images after passing through a denoising algorithm, where large PSNR values indicate little corruption of the original image. We calculated this statistic across our set of 308 images at each level of added noise. The results are shown in Fig 7 for added noise (solid black line) and denoised (circles) images for the various filter bandwidths and types used in the experiment. The set of six different coloured circles (for the 2x3 filter conditions) superimpose (and can be barely distinguished), supporting our experimental finding that the filter types and bandwidths tested were equally effective.

As the noise contrast increased, PSNR reduced with a slope of -0.5 (in dB units; note that we use different dB conversions on the x and y axes for consistency with the literature–the PSNR values are scaled by a factor of 10 (see Eq 8), and the contrast values by a factor of 20 (see Methods). If the same scaling factor were used on both axes the slope would be -1) for added noise, and a slope of around -0.25 for denoised images. We projected the empirical thresholds (in units of noise contrast) onto the x-axis of Fig 7 to estimate the PSNR that corresponds to detection thresholds from our 2AFC experiment. This is given for the baselines (black dashed line) and the average of the denoised conditions (purple dashed line). It appears that for both noisy and denoised images, human detection threshold occurred at a PSNR of around 18dB. This equivalence might serve as a useful heuristic for future studies in which it is not possible to perform behavioural experiments.

## 4. Discussion

We measured observers’ ability to detect noise added to an image, both before and after denoising using a multiscale filtering technique. The denoising algorithm removed around 6dB of perceptually salient noise, indicating a perceptually meaningful improvement in visible image quality. We also compared different filter bandwidths and filter types, but found no consistent differences between the conditions tested, either using the detection paradigm, or by calculating an error statistic (PSNR). This demonstrates that Cartesian-separable log-Gabor filters are as good as their polar-separable cousins for the present application.

### 4.1 Limitations of denoising algorithms

Standard image denoising algorithms are effective only for noise with an approximately flat (e.g., white) spectrum. This is because they reduce activity in the high spatial frequency filter bands, which will contain a disproportionate amount of noise power, but very little image information owing to the approximately 1/*f* spectrum of natural images. Noise with a markedly different spectrum, such as pink (or fractal) noise which itself has a 1/*f* spectrum, pose a much harder challenge to denoising techniques. However, most image noise, from digital camera CCD sensor noise or electromagnetic interference, for example, is approximately white. This means that filter-based denoising algorithms are useful in a wide range of situations, including medical, security and space imaging, as well as consumer photo-manipulation software.

### 4.2 The human detection process

The details of our findings here might be specific to our experimental conditions. For example, different stimulus durations, image sizes and resolutions would be expected to influence human detection performance. However, the purpose of our study was more general than this. Our aim was to show that psychophysical performance is applicable to the quality assessment of image processing algorithms, exemplified here by a denoising algorithm. We have achieved this aim.

One limitation of all psychophysical measures of performance is that while the experimenter learns what signal level can be detected reliably, the perceptual cues for doing this cannot be inferred from the data. In our case, our results cannot tell us what image distortions were being detected by the observer in the denoised images. Nonetheless, some comments are worthwhile. The spatial frequency bandwidths of our filters increased with spatial frequency (when expressed in linear units) and so it is the higher frequency filters that detect most of the noise. This causes high spatial frequency filtering artefacts when the local noise levels exceed the thresholding of the denoising algorithm, visible as distorted copies of the filter kernel superimposed on the image. On the other hand, the thresholding in the denoising algorithm can also cause a loss of legitimate image information, again at higher spatial frequencies, causing blurring. In principle, observers might use either or both these cues to perform the detection task and differences in strategy might explain some of the individual variation between observers (see Fig 6).

Regardless of the cues used, it is likely that the ability to detect added noise will be greatest at the fovea, with peripheral vision showing a greater tolerance (e.g., lower sensitivity) to both added noise and denoising artefacts. The precise content of an image or image region will also influence performance. For example, the upper right corner of the image in Fig 1E shows more salient filter artefacts than the lower left corner. For this reason, thresholds measured using the present technique are valid only for an ensemble of images and may not accurately predict performance for a single specific image.

### 4.3 The forced-choice detection paradigm

Central to our approach is that forced-choice experiments have ground truth. This allows the experimenter to learn about human ability to detect that objective truth. In the case of image processing algorithms, the main challenge for experimental design is to identify a signal for which the performance measure will pertain to image quality. We showcased the approach here in the context of a denoising paradigm because this provided an obvious pointer to the choice of external image noise as the signal. But how might the forced-choice approach be extended to other image processing algorithms, such as image compression, deblurring and edge-detection?

### 4.4 Using 2AFC to assess other types of image processing algorithm

For algorithms of the type introduced above, the removal of image noise is not the goal, so image noise might not be an obvious choice of signal. (We come back to this in Section 4.5.) Nonetheless, 2AFC performance measures are still applicable.

#### 4.4.1 Compression algorithms

The most straightforward example, and one that preceded our own work, is the use of 2AFC detection of image compression artefacts where the observer chooses between the original image and a compressed version of that image [10,11]. This provides a clean measure of the level of a particular type of compression that can be tolerated by the human visual system.

#### 4.4.2 Deblurring algorithms

Deblurring algorithms take a blurred image and attempt to restore the original unblurred image [e.g. 9]. If the depth of restoration is too light, then blur remains, if it is too heavy, then image artifacts are introduced. The approach we offer for assessing the quality of such algorithms follows a similar logic to that we used for denoising algorithms. Since denoising removes noise, we used added noise as a signal, and since deblurring removes blur, we propose using added blur as a signal. Thus, the baseline condition involves measuring a 2AFC psychometric function for the detection of blur (added to a library of images at a range of levels), and the test condition does the same but after those same images (blurred and unblurred) have been passed through the deblurring algorithm. This approach will indicate the level of blur that the deblurring algorithm can hide before either algorithm artefacts and/or blur become detectable.

#### 4.4.3 Edge detection algorithms

Edge detection algorithms take an image as input and produce an edge map of luminance boundaries as output. This is a classic example of where readers are often left to judge for themselves which algorithm is best [e.g. 8]. How can this be made into 2AFC? If an edge map is a high-quality representation of the original image, then observers should be able to readily identify it with that image. On the face of it, this is a trivial task, but observers can be pushed to the limits of their ability by superimposing ‘distractor maps’ on the stimulus. To test a specific edge detection algorithm, we suggest applying it to a library of images. A 2AFC trial would consist of a preliminary presentation of a randomly selected target image followed by a stimulus pair of *n* superimposed edge maps, each randomly selected from the library of the edge maps for other images. In one of those images, one of the maps is replaced by the target edge map, the task being to detect which one. The number of superimposed edge maps, *n*, is varied from trial-to-trial to generate a psychometric function for detection of the correct edge map.

One potential problem here is that the edge maps produced by most edge detection algorithms for natural (photographic) images are so similar that the experiment might not have the required sensitivity (i.e., integer steps of distractors might be too coarse to resolve perceptual differences between algorithms). On this matter, we suggest two possible solutions. One is to use cropped images where features are sparse and where different algorithms might perform differently. Another approach is to use a library of synthetic images (made from superimposed sine-wave gratings) designed to reveal the different mechanics of the algorithms under test [40–42]. In practice, this might need some fine tuning to be effective; our aim here is to highlight an empirical direction with potential.

### 4.5 Extending the 2AFC approach

Our approaches for denoising and deblurring above used signal level as an independent variable for measuring a psychometric function. Needless to say, the depth of algorithmic treatment could be controlled by the experimenter as a second independent variable (e.g., the threshold parameter in the denoising algorithm we used here). This would help provide a quantitative measure of when the algorithm’s artefacts exceed the benefits of what the algorithm brings. This might be particularly valuable for algorithms that contain several critical internal parameters.

The type of experiment we propose for edge detection algorithms above (Section 4.4.3) prompts another approach that might be taken with denoising and deblurring algorithms. Instead of using noise as the target, it could be used as a mask added to each image *after* the application of the algorithm as appropriate. The task would be to detect which of two images (clean versus corrupted plus algorithm) had been treated by the algorithm for variable levels of (fractal) noise mask whose spectra match the original image (i.e., phase randomized versions of the original image). In this case, the lower the added noise that can be tolerated to a criterion level of performance (e.g., 75% correct), the better the algorithm. This can be valuable for assessing algorithms intended to deal with such heavy corruption (by noise or by blur) that algorithm artefacts are expected but, for whatever reason, are preferred over the original corruption (see examples in Fig 8 of [12]). Note that this approach should work well for comparisons across algorithm since (i) severe artefacts will require high levels of fractal mask to hide them and (ii) underperforming algorithms that do not remove the original corruption will require high levels of fractal mask to hide (a) the high spatial frequency components of white image noise in the case of denoising algorithms and (b) hide the absence of high spatial frequency image components in the case of deblurring. However, once again, our aim is to highlight an empirical approach with potential. In practice, no doubt, it would need some fine tuning.

## 5. Conclusions

We have introduced and tested a new performance-based psychophysical method for assessing the efficacy of image denoising algorithms. Performance methods do not suffer from the criterion effects that undermine the subjective techniques presently found in the literature [1,43–46]. We have also suggested ways to apply the approach to other types of image processing algorithms. Finally, we provided a formal derivation of a Cartesian-separable version of the log-Gabor filter with polar parameters which offers some advantages over the traditional polar-separable version, particular for the biological vision community. This type of filter was shown to have equal merit to its polar separable equivalent for the denoising algorithm used here.

### Appendix A: Derivation of the Cartesian separable 2D log Gabor function

Our log Gabor stimuli and filter kernels are Cartesian separable with polar parameters (*f*, *θ*) and are defined in the Fourier domain. Spatial frequency bandwidth is defined as full-width at half-height in octaves (ω) at the best orientation (*θ*_0_) and orientation bandwidth is defined as the orientation half-width at half-height (*h*) at the best spatial frequency (*f*_0_). (This is commonplace in the biological vision literature.) In linear coordinates, the orthogonal 1D function (orthFunc(*f*, *θ*)) is Gaussian with a full-width at half-height, *y*. On log coordinates, the 1D log Gabor function (logGab1D(*f*, *θ*)) is Gaussian with a full-width at half-height, ω. (See Fig 7 for a sketch of the geometry).

The two-dimensional Cartesian separable log Gabor function (logGab2D(*f*, *θ*)) is the product of two 1D functions:

logGab2D(f,θ)=logGab1D(f,θ)×orthFunc(f,θ). (A1)

Equations for the two 1D functions were presented without derivation in Meese [26]. We report the derivation of a simpler form of those equations here. The standard equation for a Gaussian function (in the frequency domain) with standard deviation *σ* is:

$$G(\sigma )=exp\left(\frac{-(f-f_{0}{)}^{2}}{2\sigma^{2}}\right).$$

Converting this to octaves we replace *f—f*_*0*_ with log_2_(*f*/*f*_*0*_), and allowing for a rotation of coordinates to place the 1D function at any orientation in 2D Fourier space we replace *f* with *f*|cos(*θ* - *θ*_*0*_)|. The full-width at half-height is related to the standard deviation by the standard conversion: *ω* = *σ*/0.424. Making each of these substitutions gives:

$$logGab1D(f,\;\theta )=exp\left(\frac{-{\left\{{log}_{2}(\frac{f|cos(\theta -\theta_{0})|}{f_{0}})\right\}}^{2}}{2(0.424{\omega )}^{2}}\right),$$ (A2)

Deriving the orthogonal 1D function is a little trickier. The orientation bandwidth (*h*) is defined at the best frequency (*f*_*0*_) and so the width of the orthogonal Gaussian *y* (Fig 7) is not straightforwardly derived from *h*.

Allowing for rotation of co-ordinates as before, the orthogonal function is given by:

$$orthFunc(f,\;\theta )=exp\left(\frac{-{\left\{fsin(\theta -\theta_{0})\right\}}^{2}}{2\eta^{2}}\right),$$ (A3)

where *η* = 0.424*y*. But we want *η* in terms of *h* (and *f*_*0*_). To achieve this we observe that the point (*f*_*0*_, *θ*_*0*_ *+ h*) of the 2D function has a value of 0.5 (by definition). Thus, we have:

$$logGab2D(f_{0},\;\theta_{0}+h)=logGab1D(f_{0},\;\theta_{0}+h)\times orthFunc(f_{0},\;\theta_{0}+h)=0.5,\;$$

from which we can write:

$$orthFunc(f_{0},\;\theta_{0}+h)=0.5/logGab1D(f_{0},\;\theta_{0}+h).$$

Expanding the l.h.s. gives:

$$exp\left(\frac{-(f_{0}sin(h){)}^{2}}{2\eta^{2}}\right)=\frac{0.5}{logGab1D(f_{0},\;\theta_{0}+h)}.$$

Taking natural logs and rearranging gives:

$$\eta =f_{0}sin(h)\sqrt{-{\left[2ln(\frac{0.5}{logGab1D(f_{0},\;\theta_{0}+h)})\right]}^{-1}},$$ (A4)

which reduces by substitution to,

$$\eta =f_{0}sin(h)\sqrt{\frac{1}{ln4-{\left(\frac{{log}_{2}|cos(h)|}{0.424\omega }\right)}^{2}}}.$$ (A5)

Thus, the Cartesian separable 2D log Gabor function is given by the product of Eq A2 and Eq A3 (i.e. Eq 1), where the parameter *η* in Eq A3 is provided by Eq A4, which is in terms of *f*_*0*_ and *h*, as required. These equations produce only a single lobe of the filter in the amplitude spectrum. The symmetrical lobe can be derived by rotation of 180° about the origin, as implemented in the Matlab code in Appendix C. Furthermore, t he phase component is set directly in the phase spectrum (the angular component of the Fourier transform) before inverse transforming back into the spatial domain.

Note that the spatial frequency bandwidth puts a constraint on the range of orientation bandwidths available. For example, as the point (*f*_*0*_, *θ*_*0*_ *+ h*) is rotated further anticlockwise in Fig 7 (increasing the orientation bandwidth) there comes a point where logGab1D(*f*, *θ*) becomes less than 0.5. When this happens, there is no value of *y* that can lift logGab2D(*f*, *θ*) to half-height at that point.

### Appendix B: Numerical analysis of maximum filter bandwidths

**Fig 8:**
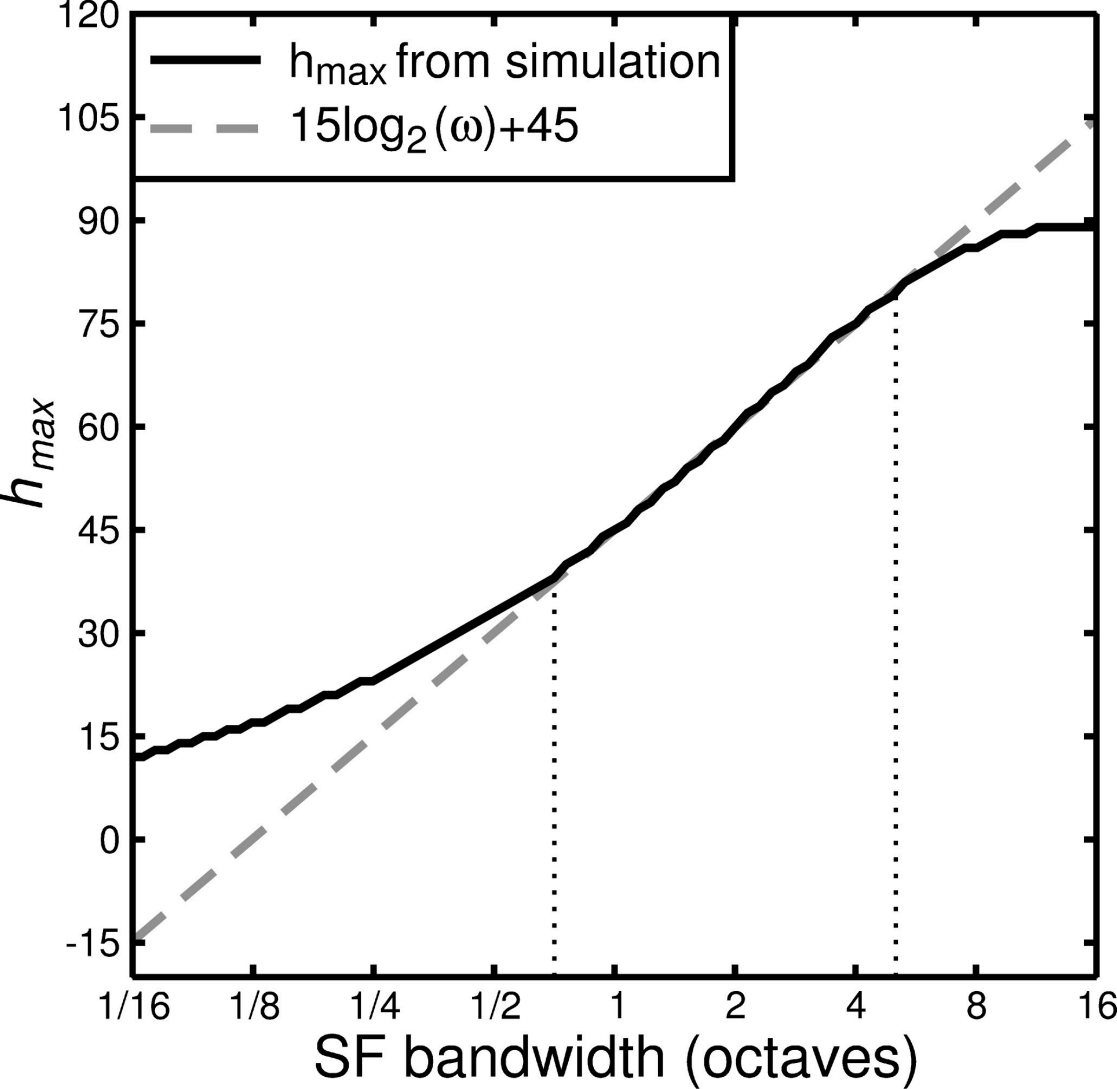
Maximum orientation bandwidth as a function of spatial frequency bandwidth (*ω*). The straight dashed line is given by 15log_2_(*ω*) + 45.

We determined the upper orientation bandwidth limit as a function of spatial frequency bandwidth by numerical analysis. Cartesian-separable log-Gabor filters were generated across a wide range of bandwidths. We assessed the point at which the area of the filter’s footprint in the Fourier spectrum stopped increasing as a function of orientation bandwidth. This corresponded to the point at which Eq A4 was undefined. These maximum bandwidths are linear as a function of log spatial frequency bandwidth over a wide range (0.7 to 5 octaves) as shown in Fig 8. We fitted a straight line to this portion of the curve, which is given by 15 log_2_(*ω*)+45. We confirmed that these limits are constant across different centre spatial frequencies, image sizes and resolutions.

### Appendix C: Example Matlab code for generating log-Gabor functions

function imLG = makeloggabor(imSize,f0,theta0,omega,h,phi,logGabType)

% imLG = makeloggabor(imSize,f0,theta0,omega,h,logGabType)

% Produces either a Cartesian- or a polar-separable log-Gabor element.

% Input args:    imSize = width and height of output image

%    f0 = centre spatial frequency in cycles/image

%    theta0 = centre orientation in degrees

%    omega = spatial frequency bandwidth in octaves (FWHH)

%    h = orientation bandwidth in degrees (HWHH)

%    phi = phase angle in degrees

%    logGabType = `c’ or `p’ for Cartesian/polar-separable

% Output:    imLG = log Gabor filter element (imSize x imSize)

% See Baker, Summers, Baldwin & Meese (2022), PLoS ONE, doi: 10.1371/journal.pone.0267056

% Distributed under the Creative Commons Attribution-ShareAlike 4.0 International license

theta0 = theta0*pi/180; % convert all angular parameters to radians

h = h*pi/180;

phi = phi*pi/180;

u = meshgrid(1:imSize,1:imSize)—((imSize+2)/2); % set up coordinates

v = u’;

f = sqrt(u.^2 + v.^2); % radial (spatial frequency) coordinate

theta = atan2(v,u); % angular (orientation) coordinate

uft = f.* cos(theta-theta0);

switch logGabType

    case ‘c’ % Cartesian-separable log Gabor

        numer = -(log2((f.*abs(cos(theta-theta0)))./(f0)).^2);

        denom = 2*(0.424*omega)^2;

        logGab1D = exp(numer./denom); % Implementation of Eq 2

        k = (log2(abs(cos(h)))/(0.424*omega))^2;

        eta = f0*sin(h)*sqrt(1/(log(4)—k)); % Eq 4

        orthFunc = exp((-(f.*sin(theta-theta0)).^2)./(2 * eta^2)); % Eq 3

    case ‘p’ % polar-separable log Gabor

        logGab1D = exp((-(log(f./f0)).^2)./(2*log(omega)^2)); % Eq 5

        sinDiff = sin(theta)*cos(theta0)-cos(theta)*sin(theta0);

        cosDiff = cos(theta)*cos(theta0)+sin(theta)*sin(theta0);

        thetaDiff = abs(atan2(sinDiff,cosDiff));

        orthFunc = exp((-thetaDiff.^2)./(2*h^2)); % Eq 6

        orthFunc = orthFunc + rot90(orthFunc,2);

end

logGab2D = logGab1D.* orthFunc; % combine the two filter components

cx1 = ones(imSize).* complex(0,0); % adjust the log-Gabor to be

cx2 = ones(imSize).* complex(0,0); % in the requested phase

cx1(uft>0) = complex(logGab2D(uft>0).*sin(phi),-logGab2D(uft>0).*cos(phi));

cx2(uft<0) = complex(logGab2D(uft<0).*sin(phi),logGab2D(uft<0).*cos(phi));

cxLogGab2D = cx1 + cx2;

realImage = fftshift(real(ifft2(fftshift(cxLogGab2D))));

imLG = realImage./ max(abs(realImage(:)));

% Filters are individually peak-normalised in this script. If generating

% multiple filters, you may wish to remove this line and rescale to the

% global peak of the filter bank, to balance power across all filters.

end

## Supporting information

S1 File Enlarged example images. (DOCX)
