# Supplementary material for: A psychophysical performance-based approach to the quality assessment of image processing algorithms
Source: PLoS One. 2022 May 5;17(5):e0267056. doi: 10.1371/journal.pone.0267056 (PMC9071145; doi:10.1371/journal.pone.0267056)
Supplement: S1 File — (DOCX) [file pone.0267056.s001.docx]

**S1 File. Enlarged example images.**


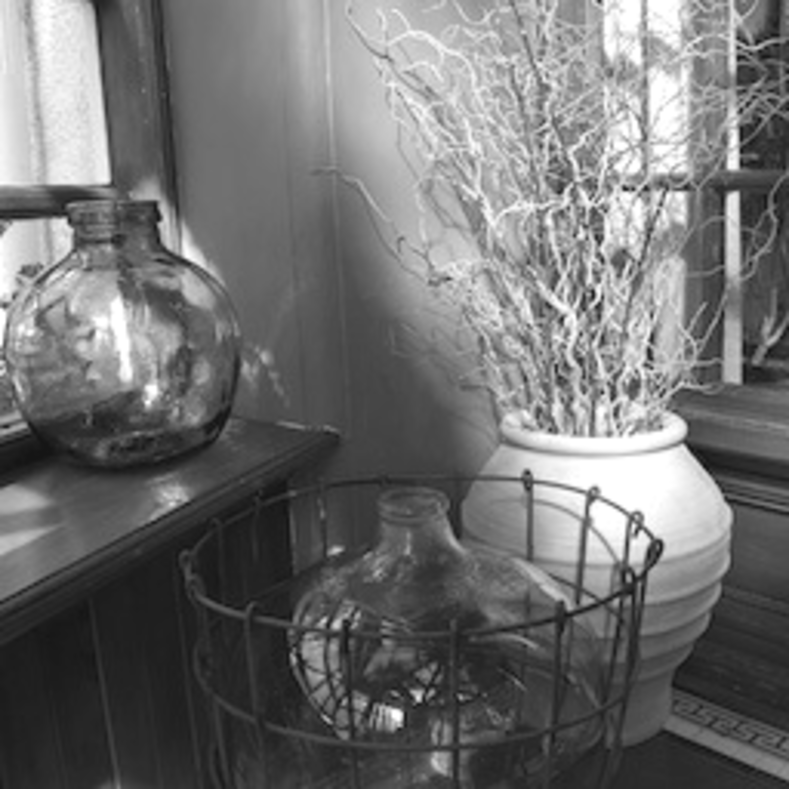


**Fig A. Original image.**

**
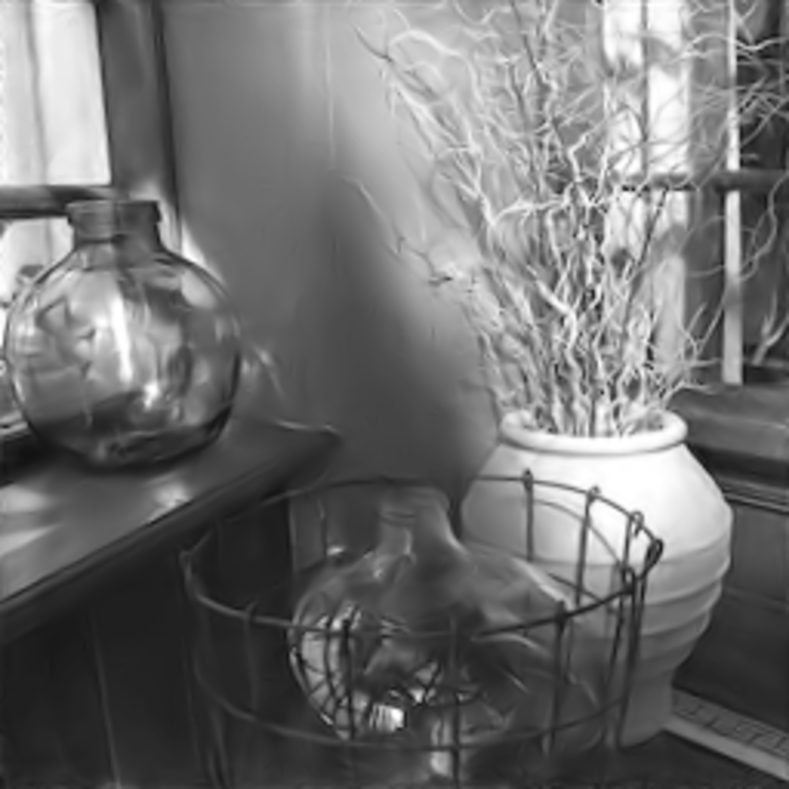
**

**Fig B. Denoised image (8% noise).**

**
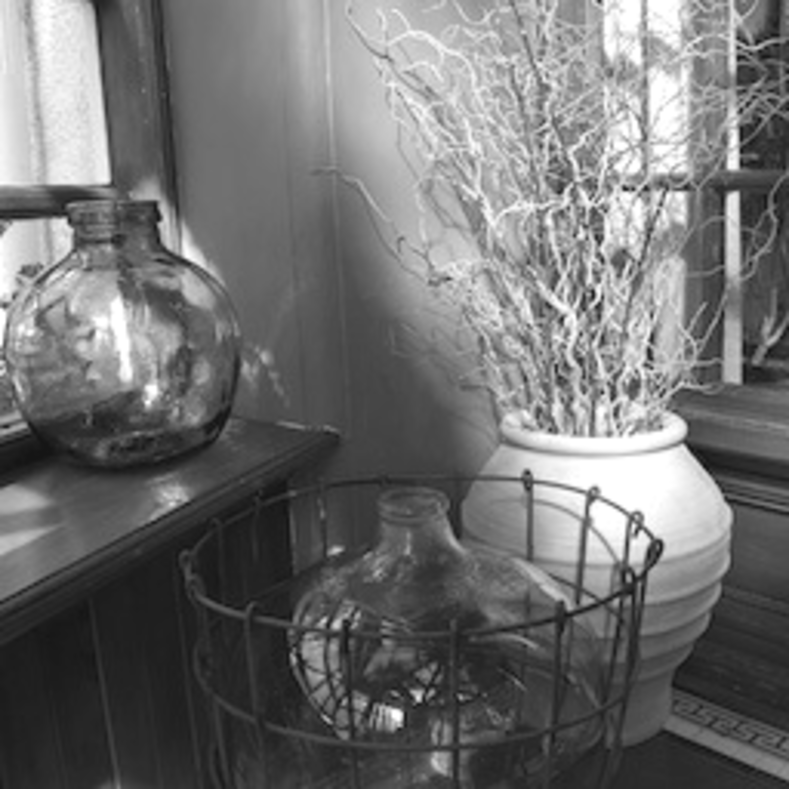
**

**Fig C. Original image.**

**
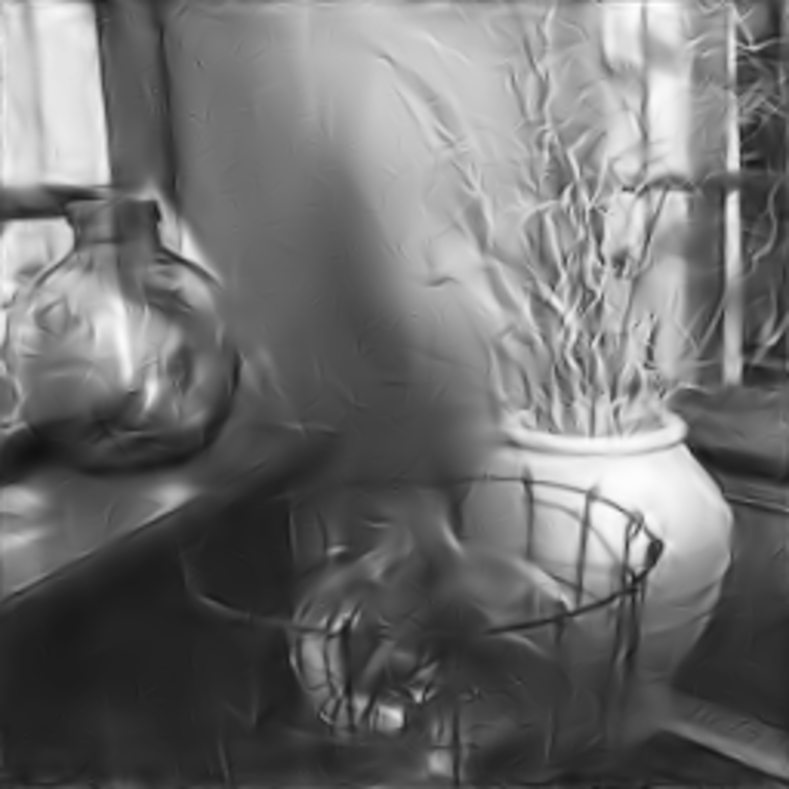
**

**Fig D. Denoised image (22% noise).**
